# Advances in Strategies to Transport Nanoparticles Across the Blood–Brain Barrier for Drug Delivery into the Brain for the Treatment of Alzheimer’s Disease

**DOI:** 10.3390/ph19050685

**Published:** 2026-04-27

**Authors:** Rafael Silva, Joana Monteiro, Maria João Ramalho, Stéphanie Andrade, Joana A. Loureiro, Maria Carmo Pereira

**Affiliations:** 1 LEPABE—Laboratory for Process Engineering, Environment, Biotechnology and Energy, Faculty of Engineering, University of Porto, Rua Dr. Roberto Frias, 4200-465 Porto, Portugal; up202004988@edu.fe.up.pt (R.S.); up202104587@edu.fe.up.pt (J.M.); mjramalho@fe.up.pt (M.J.R.); stephanie@fe.up.pt (S.A.); 2 ALiCE—Associate Laboratory in Chemical Engineering, Faculty of Engineering, University of Porto, Rua Dr. Roberto Frias, 4200-465 Porto, Portugal; 3 Department of Mechanical Engineering, Faculty of Engineering, University of Porto, Rua Dr. Roberto Frias, 4200-465 Porto, Portugal

**Keywords:** neurodegenerative diseases, biological barriers, nanoparticles, drug delivery systems, nanotechnology-based strategies, clinical translation

## Abstract

Alzheimer’s disease (AD) is a prevalent neurodegenerative disorder characterized by progressive dementia, constituting one of the leading causes of global mortality. Although the current treatments help attenuate the symptoms associated with AD, they are unable to stop the long-term progression of the disease, and consequently, no cure exists. One of the main reasons for the lack of cure and, therefore, one of the biggest challenges in its treatment, is the blood–brain barrier (BBB). This protective barrier limits the entry of foreign substances, including drugs, into the central nervous system. Different types of engineered nanoparticles (NPs) have been demonstrated to be able to penetrate this barrier and serve as efficient drug delivery systems (DDS) into the brain, making them a promising solution for future therapeutic development. Therefore, the purpose of this paper is to provide valuable insights into challenges faced by DDS in treating AD, highlight the nanotechnology-based approach, and discuss the advances in strategies being employed to enhance the crossing of NPs through the BBB. Furthermore, some up-to-date NP systems are presented, along with the latest therapeutic agents targeting AD, and finally, it underscores innovative approaches under investigation. Ultimately, the barriers hindering the clinical translation of NP-based strategies into human patients are discussed.

## 1. Introduction

Neurodegenerative diseases (NDs) are traditionally defined as disorders with selective and symmetric loss of neurons in motor, sensory, or cognitive systems and distinct involvement of functional systems, defining clinical presentation [1]. Some of the most common NDs are Alzheimer’s Disease (AD), Parkinson’s disease, spinal system atrophy, Huntington’s disease, amyotrophic lateral sclerosis, motor neuron disease, and spinocerebellar ataxia [2].

AD is the most prevalent form of dementia, constituting, therefore, one of the leading causes of global mortality, with crescent growth. Coupled with the inherent socioeconomic impact derived from the health care system, there is an urgent need for effective therapeutic solutions [3]. This multifactorial disease, whose origin may stem from aging, environmental factors, lifestyles, comorbidities, and even genetic predisposition, is accompanied by several pathological hallmarks, highlighting the abnormal accumulation of beta-amyloid (Aβ) and neurofibrillary tangles (NFTs). However, the heterogeneity of these hallmarks cannot be fully comprehended in the light of a single hypothesis [4].

The lack of therapies for AD and other NDs is primarily a result of the blood–brain barrier (BBB). The central nervous system (CNS) has different barriers that protect it from several substances. These barriers include the blood–cerebrospinal fluid (blood–CSF) barrier, the blood–retinal barrier, the blood–spinal cord barrier, and the BBB. Among these, the latter is the most extensive and exclusive [5]. It is a highly selective semipermeable barrier that regulates the uptake and release of substances into the brain, responsible for the protection against pathogenic molecules [6].

Nanotechnology integrated in drug delivery systems (DDS) offers a wide array of solutions based on nanoparticles (NPs). These systems offer an enhanced value due to high stability, loading capacity, and bioavailability, protection against enzymatic degradation, and creation of a sustained and localized release. In this way, they are proposed as an effective alternative to cross the BBB once their surface can be easily engineered to circumvent this biological barrier. Additionally, they can overcome the free drug’s limitations, like a lack of selectivity, and reduce the risk of adverse secondary effects by decreasing the drug’s administered concentration [7,8]. Therefore, they have proven to be highly relevant in the AD context as they provide a protective vehicle to carry and deliver therapeutic agents to the brain safely [8].

For this review, a comprehensive literature search was conducted using scientific databases, such as PubMed and Google Scholar. General keywords such as nanoparticles, Alzheimer’s disease, in vitro studies, in vivo studies, and encapsulated drug were initially used. For more specific research, additional keywords were included, such as surface functionalization, stimuli-responsive, focused ultrasound, and magnetic nanoparticles. Articles were selected based on the year they were published, preferably articles published after 2020; the inclusion of in vitro and/or in vivo studies; direct investigation of the BBB and/or AD; and presentation of positive or relevant experimental results.

## 2. Challenges in Drug Delivery to the Brain

Although of the utmost importance for the existence of a stable and safe environment and the maintenance of its homeostasis, the BBB limits the action of drugs in the brain [6]. This barrier is responsible for the regulation of molecule influx and efflux into and out of the brain. This function is ensured by a tightly regulated neurovascular unit composed mainly of endothelial cells (ECs) sealed by tight junctions (TJs) and structurally sustained by the basement membrane, pericytes, and glial cells. Among the latter, astroglia (astrocytes) and microglia are particularly relevant [9].

**Figure 1:**
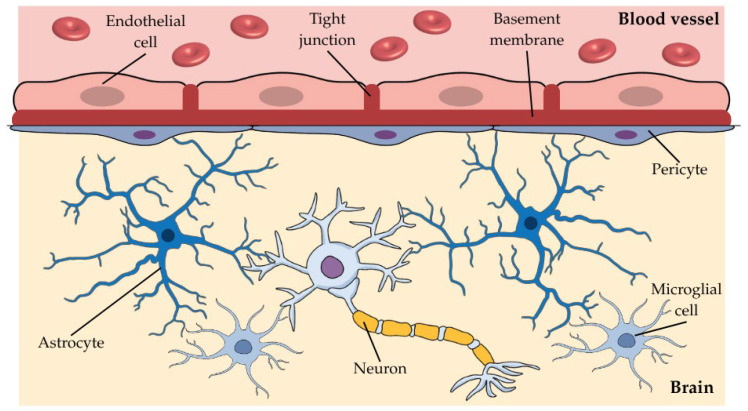
Main components of the BBB. This image was created with the software Canvas (https://www.canva.com/).

ECs are the core anatomical structure of the BBB responsible for the link between the blood vessels and the CNS. Their membrane is negatively charged and allows the passage of small lipophilic molecules and specific substances via transporters present in their surface [6]. ECs are highly sealed by TJs that impede the paracellular diffusion of most molecules, besides causing blood vessels to have high transendothelial electrical resistance, which translates to the integrity of the cellular barriers in restraining the passage of substances into the brain [6,10]. Additionally, the basement membrane has structural, functional, regulatory, and mediator properties, whilst pericytes have a key function in maintaining the effective performance of the BBB through the connection with the ECs, controlling its permeability, angiogenesis (ability to form new blood vessels from existing ones), among other functions. Finally, astrocytes that, despite not displaying permeability attributes, have a crucial role in dynamic signaling and microglia have protective properties to maintain a healthy environment [9,11]. These core elements are represented in Figure 1.

Therefore, the high selectivity of the BBB can be attributed to physical (highlighting TJs to prevent paracellular diffusion), transport (efflux pumps), and metabolic (enzymes to inactivate molecules) barriers. Together, they form a symbiotic relation to protect the brain from harmful substances [12,13].

### Transport Mechanisms Across the BBB

Due to this selectivity, drug delivery into the brain requires the development of specific technologies to surpass this barrier. These can be classified into three main categories: circumventing the BBB by either invasive or non-invasive routes, disruption of the BBB and passage across the BBB, often mediated by receptors and transporters.

Circumvention of the BBB via local routes can involve invasive procedures, such as intracerebroventricular and intrathecal ones, that deliver drugs directly into the CSF or brain tissue but remain inefficient and clinically restricted [14]. In contrast, a non-invasive approach such as intranasal administration offers a direct delivery through the olfactory or trigeminal nerve pathways, although some drugs may also enter systemic or lymphatic circulations, requiring crossing the BBB and limiting the amount that reaches the brain. In practice, both pathways occur. Advantages of this type of administration include high patient compliance, avoidance of hepatic first-pass metabolism and intestinal enzymatic degradation, evasion of BBB penetration, and rapid delivery kinetics. The main challenges related to intranasal administration lie in the transition from in vitro and animal models to humans due to the anatomical differences in the nasal cavity [15]. This creates an urgent need for the development of realistic 3D models, as well as for the evaluation of the safety of excipients and adjuvants besides the drug itself. This is relevant, given the low brain-targeting efficiency and route-dependent disease selectivity, which still require further studies to be validated [15].

Disrupting the BBB, which consists of opening the TJs of the brain using non-invasive physical methods like infusion of noxious agents (e.g., bradykinin that induces vasodilation) or injection of microbubbles (MBs) (small vehicles filled with gas), followed by sonication (e.g., focused ultrasound (FUS) sonication), is another strategy [16]. However, prolonged BBB disruption associated with long-term treatment raises safety concerns, as it may facilitate neurotoxic effects in the brain [14].

Finally, some molecules can cross the BBB by two main pathways, transcellular or paracellular, through the cell or the intercellular space, respectively [14]. Small lipophilic molecules, generally with a molecular weight under 400 Da and less than 8 hydrogen bonds, and gases, such as O_2_ and CO_2_, can easily cross the ECs and reach the brain, defining the transcellular diffusion [17]. Also, despite the TJs that seal the ECs, some small and soluble molecules are also able to cross this gap between cells without mediation (paracellular pathway) [6]. However, the rest of the molecules, essential for brain metabolism, that do not possess those features and, therefore, are not capable of reaching the brain on their own, need to resort to the aid of mediators. Carrier-mediated transport (CMT) refers to the transport of a variety of molecules facilitated by membrane-bound proteins entitled solute carrier (SLC) transporters [5]. Examples of those include the glucose transporters (GLUTs) responsible for the transport of glucose for energy purposes [18]. The following two mechanisms involve the transcytosis of larger molecules from one side to the other of the ECs, since their size limits the use of SLC transporters. In the adsorptive-mediated transcytosis (AMT), positively charged molecules interact with the negatively charged ECs, inducing transcytosis and their passage across the barrier. On the other side, in the receptor-mediated transcytosis (RMT), specific molecules bind to their respective receptors present on the surface of these cells, triggering transcytosis, and their release to the brain environment by detaching from the receptor [5]. Examples of receptors present in the BBB include the transferrin receptor (TfR) and the insulin receptor (IR), responsible for iron and glucose uptake by the binding of transferrin (Tf) and insulin, respectively [18]. Contrary to the other pathways, the active efflux is responsible for the expulsion of some molecules from the brain to the blood circulation via ATP-binding cassette (ABC) transporters [17]. In the AD context, from the 48 ABC transporters identified, the P-glycoprotein (P-gp) is recognized as one of the Aβ efflux pumps [19]. Impaired function of these types of transporters accelerates AD progression by limiting Aβ clearance and promoting its accumulation in the brain [20].

**Figure 2:**
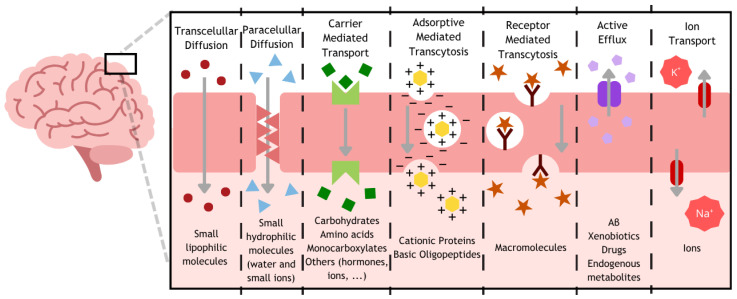
Potential pathways across the BBB. This image was created with the software Canvas (https://www.canva.com/).

Finally, ion transport is also a suitable strategy. Ion channels are essential to sustain a healthy brain function through processes such as the release of neurotransmitters, the generation of action potentials, and the enabling of synaptic plasticity, among others [21]. An example of an ion channel is the Na^+^-K^+^-ATPase that regulates the influx of sodium and efflux of potassium to maintain an ion gradient across the membrane, whose principal roles consist of the uptake and release of neurotransmitters and cellular excitability [22]. All these mechanisms are represented in Figure 2.

## 3. Physicochemical Properties Influencing Nanoparticle Passage Across the BBB

Many types of NPs have been shown to enhance the targeting efficiency of drugs to the brain and reduce their adverse effects caused by the systemic distribution. However, several parameters affect the efficiency of the NPs as brain DDS, determining whether they are suitable to cross the BBB. These parameters include size, polydispersity index (PdI), zeta potential (ZP), shape and chemical composition. Small NPs (<30 nm) are often eliminated via renal excretion, while larger ones (150–300 nm) tend to accumulate in organs like the liver and spleen [13]. Studies assure the safety of NPs with diameters above 100 nm [23]; however, to be able to cross the BBB, the literature indicates that NPs should be smaller than 200 nm [24]. As such, the appropriate size range for DDS is between 100 and 200 nm.

NPs’ stabilization is achieved by repulsive interactions like electrostatic or steric forces that prevent aggregation. To determine a particle’s electrostatic charge and assess its stability, the ZP is commonly measured [25]. It measures the potential in a diffusive layer that involves the Stern’s layer (the first ion layer formed on the NPs’ surface) under the application of an electrical field [26]. The ZP is affected by a set of factors such as suspension’s concentration of NPs, pH of the medium, temperature, ionic forces, among others. The stability range of a suspension usually corresponds to ZP above 25 mV or under −25 mV. However, this interval is variable, and each case must be evaluated separately to determine if a solution is stable or not [25].

In the context of NDs, NPs with high positive ZP can present toxicity to the BBB, since, as previously mentioned, the BBB endothelium carries a negative surface charge. Such electrostatic attraction between the two interfaces (NPs and BBB) leads to the disruption of TJs, and ultimately, the opening or dysfunction of the barrier [27]. As a result, most of the NPs studied for brain delivery have between moderate (−1 mV to −15 mV) and high (−15 mV to –45 mV) negative ZP [28]. However, neutral NPs have a higher ability to permeate the BBB without affecting its integrity and reduce protein adsorption, leading to a longer circulation time, making them the most suitable to be used as brain DDS [29].

Regarding the surface’s hydrophobicity, the usage of surfactants like polyvinyl alcohol (PVA) contributes with steric repulsions, responsible for the increase in stability and prevention of aggregation, allowing, therefore, control of the NPs’ size [30].

The shape of the NPs is another critical parameter influencing their efficiency as DDS. Different shapes result in various surface areas/volume ratios, which can consequently affect cellular uptake, biodistribution, and viability [31,32].

Studies also investigated the effect of shape on the transcytosis of the BBB. A study using a multicellular 3D spheroid model of the BBB investigated gold NPs (AuNPs) with two different morphologies, spherical and rod-like. The results indicated that spherical AuNPs, although showing limited translocation through the endothelial barrier, penetrated more deeply into the interior of the spheroid once internalized. Rod-shaped AuNPs exhibited a higher initial capacity to cross the BBB but tended to accumulate near the spheroid’s periphery, with restricted penetration into its core [33]. Another study, using in vitro Transwell models, showed that nonspherical NPs exhibit reduced transport compared to their spherical 200 nm diameter counterparts, suggesting that transcytosis could be affected by the shape [34]. These findings highlight the critical role of shape in NP-mediated BBB. Consequently, by tailoring NPs’ shape, researchers can enhance transcytosis across the BBB, making shape another crucial parameter to consider during NPs design and production [32,35].

Lastly, the chemical composition of the NPs can also influence their ability to cross the BBB, as it plays a crucial role in biocompatibility, biodegradability, and potential toxicity [36]. Since their discovery, liposomes have been the most common DDS studied for a broad number of drugs [37]. Because liposomes are made with lipids, they are biocompatible, biodegradable, and can fuse to cell membranes. Additionally, due to their size, fluidity, permeability, and composition, they are suitable for controlled drug release, while being versatile in the drug encapsulation, as they can encapsulate both lipophilic and hydrophilic drugs (dual solubility) [14]. Polymeric nanoparticles (PNPs) are also highly used as DDS. They are organic-based NPs composed of polymers from either natural (e.g., chitosan) or synthetic (e.g., poly(ε-caprolactone) (PCL)) origin and can be biodegradable (e.g., poly(d,l-lactic-co-glycolic acid) (PLGA)) or not (e.g., polyacrylates) [38]. Beyond the properties entailed to NPs, PNPs can have positive or negative charge, depending on the polymer, a high drug loading capacity, and the ability to be easily tailored with non-ionic surfactants during formulation for stealth purposes against opsonization and specific ligands to improve brain targeting. Among the different polymers, PLGA is the most used [23,39]. PLGA NPs’ popularity is attributed to their unique particularities: controlled and sustained release profile, low toxicity, biocompatibility with cells and tissues, easy production, and approval by the Food and Drug Administration (FDA) and European Medicines Agency (EMA) [14,23]. Therefore, NPs’ chemical composition can vary, and selecting an appropriate material is essential to ensure both efficient brain delivery and minimal adverse effects.

## 4. Nanoparticle-Based Drug Delivery for Alzheimer’s Disease Treatment

The evidence found by postmortem brain tissue analysis, neuroimaging technology, CSF content, and animal research strongly supports a connection between BBB dysfunction and AD [40]. This cognitive dysfunction was even discovered to predate by several years the mild cognitive impairment (MCI) phase, where the first symptoms start to arise. BBB breakdown is categorized by a cascade of structural and functional alterations impairing its function in preventing the entry of foreign substances into the CNS, therefore compromising neurons’ integrity and triggering neurodegeneration [40]. These can include increased paracellular permeability by loss of TJs, degradation of ECs and pericytes, modification in thickness of the basement membrane, astrocyte degeneration, changes in the expression of transporters/receptors present in the BBB, and so forth [41].

AD’s pathogenic factors are central to BBB damage. The dysregulation of receptors responsible for Aβ homeostasis leads to Aβ aggregation in the vessel walls (amyloid angiopathy), impairing the BBB function and worsening vascular pathology. NFTs exacerbate BBB damage. The barrier’s dysfunction impedes the clearance of oligomeric tau proteins and promotes tau diffusion. Additionally, as a response to inflammation, CNS glial cells produce proinflammatory cytokines that compromise the barrier’s integrity, preventing its repair and, consequently, exacerbating inflammation. These mechanisms are often perceived as a “vicious cycle”: these alterations initiate BBB breakdown that disrupts the brain’s normal activity, triggering progressive synaptic and neuronal dysfunction, which in turn further compromises the barrier [40].

The BBB breakdown not only contributes to AD progression but also severely limits the effective delivery of therapeutic agents to the brain. Interestingly, even unmodified NPs have an intrinsic ability to surpass BBB restrictions. In a study conducted by Buzulukov et al. [42], Ag NPs were able to reach the brain tissue (66.4 ± 5.6 ng of Ag detected in the brain from a dose of 0.1 mg/kg of body weight (BW) for 28 or 14 days). Therefore, since their baseline design already proves this capability, advanced strategies may further amplify their internalization and performance.

### 4.1. Surface-Functionalized Nanoparticles

Active targeting implies the adhesion of ligands to the NPs’ surface to specifically direct them to the action site. These functionalized vehicles are frequently labeled as “Trojan horses”, derived from the Greek mythology [11]. In this way, transcytosis modifications grant the NPs’ surface with ligands for BBB recognition and enable brain internalization. Post-transcytosis modifications allow for even further specification, orienting the NPs directly to the site of action. The choice of the ligand is crucial, as it must have affinity to receptors/transporters present at the BBB and be in sufficient number. However, saturation of these ligands may limit efficiency by causing steric hindrance, size increase, and impair exocytosis, among others. Therefore, optimizing ligand concentration is essential [43].

**Figure 3:**
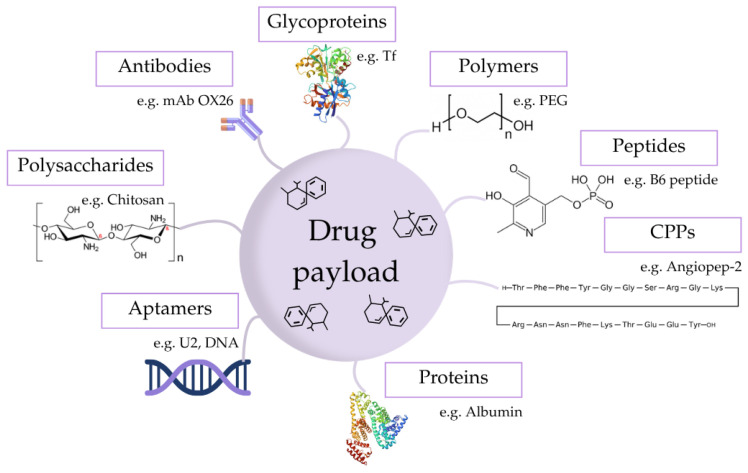
Scheme of types of molecules commonly used for NP surface modification. This image was created with the software Canvas (https://www.canva.com/). Tf and albumin structures (pdb_00001a8e and pdb_00001ao6, respectively) were retrieved from RCSB PDB (https://www.rcsb.org/).

Modifying the surface of the NPs with specific ligands (e.g., polymers, proteins, antibodies, peptides, aptamers, etc.) is a suitable strategy to enhance their performance in transporting drugs across the brain and improve their therapeutic efficiency. The functionalization can be categorized into pre-transcytosis (before crossing the BBB cells) to increase the half-life’s circulation time in the blood; transcytosis to improve the passage across the BBB; and post-transcytosis to direct the NPs to the damaged nerve cells [30]. Table 1 presents an array of ligands and surface coating molecules of different natures for NP modification, properly categorized, while Figure 3 displays a schematic representation of ligand decorations.

Note that not all the ligands are suitable for all different types of NPs. Tests should be performed to determine if their coating is feasible and if they are effectively displayed on the NPs’ surface.

Surface functionalization can be achieved by multiple strategies, such as chemisorption and physisorption, electrostatic, covalent, and non-covalent (such as hydrogen bonding, π-π interactions, van der Waals interactions, among others) interactions, and intrinsic surface engineering [61]. The use of surfactants such as PVA during NP formulation, where high shear stress forces (e.g., sonication and agitation) are applied to the sample, fits in the physisorption strategy. For the covalent conjugation, typically one resorts to organofunctional alkoxysilanes, N-hydroxysuccinimide (NHS), glutaraldehyde (GA), and 1-ethyl-3-(3-dimethylaminopropyl)carbodiimide (EDC) [61]. For example, EDC-NHS coupling allows reactive NHS esters to covalently bond with the primary amine group of a peptide, allowing the formation of peptide-conjugated PLGA NPs [30].

As the most common strategy for pre-transcytosis, PEGylation can be accomplished by covalent conjugation, physical adsorption, and self-assembly. Once physical adsorption translates into weaker interactions, often resulting in the detachment from the NPs’ surface, covalent binding is preferred. However, PEG itself cannot strongly bind to the surface because it lacks functional groups (biologically inert), demanding its combination with other molecules [62]. The most appropriate PEG derivative varies depending on the type of NP. For example, PEG-PLGA NPs can be produced either by blending PLGA with PLGA-PEG copolymer or by using amine-PEG and coupling through the PLGA carboxylic groups, resorting to EDC/NHS, whilst thiol-PEG-amine is used to attach carboxylic groups to gold NPs and silane-PEG-carboxylic for amine groups in silica NPs [46,63]. PEGylated NPs produced by self-assembly are obtained via two methods: nanoprecipitation and emulsification. Kesharwani et al. [64] fabricated PEG-PLGA NPs by mixing both polymers in an organic solvent. This mixture was then emulsified with an aqueous solution containing a surfactant and submitted to high-shear stress forces through sonication or agitation to form an oil-in-water emulsion. After organic solvent evaporation, solid NPs were obtained.

To provide an overview of some examples of surface-modified NPs currently being explored for drug delivery in AD, Table 2 presents nanocarrier formulations tested in vitro and/or in vivo. The table also highlights the diversity of nanocarrier matrices and therapeutic agents being encapsulated, as well as administration routes, test models used, and the main results obtained.

Kong et al. [49] produced osthole-loaded Tf-modified liposomes with a particle size of 104.28 ± 3.76 nm, adequate for BBB crossing, a PdI of 0.194 ± 0.009 (<0.2), indicating a monodisperse sample, and a ZP of −6.95 ± 0.56 mV, a moderate negative value. Atomic force microscope (AFM) images revealed spherical structures with smooth surfaces. As expected, Tf modification enhanced BBB transport by RMT. Osthole is a natural coumarin derivative with anti-inflammatory, anti-oxidative, and anti-apoptotic effects, but lacks stability, solubility, bioavailability, and has low brain-targeting capacity. Therefore, it presents as a potential candidate for DDS to surpass its drawbacks that prevent its usage alone. In vivo experiments were conducted by administering intraperitoneally 10 mg/kg of phosphate-buffered saline (PBS) (control), osthole solution, osthole-loaded liposomes, and osthole-loaded Tf-modified liposomes, after which the animals were sacrificed.

Arora et al. [65] perceived BDNF as a vital neurotrophin due to its role in neuronal function, cell proliferation, and synaptic plasticity, and conceptualized its delivery via plasmids since the level of this protein is reduced in AD patients. For that, they would be preserved in dual-functionalized liposomes, both to improve BBB passage: MAN and CPPs (rabies virus-derived peptide (RDP) or penetratin (Pen)). Pen-MAN and RDP-MAN liposomes had a particle size of 181.6 ± 6.14 and 178.6 ± 0.91 nm, a ZP of +18.0 ± 2.26 and +19.5 ± 2.12 mV, and a PdI of 0.208 ± 0.01 and 0.302 ± 0.01, respectively. Despite the positive values of ZP, no adverse effects were reported. The plasmids encoding BDNF and PBS (control) were injected intravenously in four doses of 0.4 mg/kg BW incorporated in liposomal nanoparticles, for the first (~15.2 nmol phospholipids/g BW), once per week.

Lin et al. [66] designed a multidrug therapy with rapamycin for enhancing autophagy and reducing AD pathological proteins and TPPU to prevent glial cell reactivation and alleviate cognitive deficits derived from AD. Both were entrapped within hybrid liposomes functionalized with platelet and CCR2-overexpressing membranes for increased circulation time and targeted intervention in inflammation sites, hereby exemplifying pre- and post-transcytosis modifications. The dual-loaded and dual-functionalized liposomes exhibited a size close to 70 nm, a ZP of ~ −7.5 mV, and a vesicle structure visualized by transmission electron microscopy (TEM). No information regarding the dosage usage for in vivo testing was reported, besides the biodistribution (1 mg/kg of DiR-labeled hybrid cell membranes via tail vein injection).

Henningfield et al. [67], instead of encapsulating a therapeutic molecule, attached a dendranib that inhibits CSF1R tyrosine kinase to the dendrimers’ surface, D-45113. The inhibition of CSF1R, present in all microglia, results in microglia death, inhibiting plaque development, and rescuing synapse and neuronal numbers. Moreover, it has been shown that it attenuates neuroinflammation, rescues synaptic integrity and cognition, and reduces tau levels. For the in vivo studies, the AD mouse model was systemically administered twice a week for 4 weeks with 200 mg/kg of the DDS described.

In this case, too, Zhong et al. [53] did not resort to encapsulating therapeutic molecules. Their team used Prussian blue in the formulation of the NPs due to its antioxidant effects (it is a ROS sequestering agent). Angiopep-2 modification, as detailed in Table 1, targets LRP-1 to ease BBB transversal. The resulting NPs had a hydrodynamic diameter of ~49 ± 15 nm, a ZP of +19 mV, and a spherical structure. Although the atypical values of the physicochemical properties, they succeed in their task. For the animal experiments, three groups were formed by the injection once a week for four weeks of 0.2 mL of saline, 0.2 mL of 0.002 mg/mL of the DDS, and 0.2 mL of 1 mg/kg of donepezil, a commonly prescribed medication to combat AD.

Pan et al. [68] identified analogous functions in LRP1. Whilst low levels of endothelial LRP1, almost absent in AD patients, contribute to BBB impairment, neurodegeneration, and cognitive deficits, high levels of neuronal LRP1 can exacerbate Aβ plaque deposition since its Aβ production mechanisms outweigh its clearance. Simvastatin is a drug able to upregulate LRP1 expression. In this way, they proposed the specific delivery of this drug to ECs via NP encapsulation by functionalizing it with Angiopep-2. Dynamic light scattering (DLS) measurements obtained an average hydrodynamic diameter of 104 nm, while TEM presented lower values, as expected. The value of the ZP was −3.6 mV, and the NPs possessed a spherical morphology. Mice were injected with 100 mg NPs/kg at the 12th and 24th hours, after which they were sacrificed.

Both the loaded therapeutic (rapamycin) and the surface functionalized (Tf) molecules’ rationale used in the NLCs designed by Khonsari et al. [69] have already been explored. Physicochemical characterization revealed spherical, with smooth surface, NPs with a particle size of 150 ± 9 nm, PdI of 0.29 ± 0.05, and ZP of +4.0 ± 1.5 mV. In the animal studies, five groups were formed, highlighting the interest in one where 3.5 mg/kg of rapamycin loaded into Tf-decorated NLCs were intrahippocampally injected.

Chen et al. [70] targeted LRP1 with mid-avidity to bias trafficking toward PACSIN2-mediated transcytosis to promote LRP1 upregulation. At the same time, it enhances Aβ clearance and reverses vascular dysfunction by means of using only their nanoformulation without the need for a therapeutic molecule. Additionally, they coated the surface with Angiopep-2 for targeting purposes. AD mouse models were injected with 0.2 mL of the formulation alongside four control treatments, and after they were culled for Aβ levels measurement, whilst for cognitive tests, they were daily injected for three days.

Guo et al. [71] developed a mesoporous nano-selenium that, by itself, reduces Aβ deposition and improves neurotoxicity. Additionally, metformin loading enhances the therapeutic potential of the DDS since it can increase the phagocytosis and Aβ scavenging ability of microglia, polarize microglia to M2 type, among other benefits. Normally, metformin is prescribed to treat type 2 diabetes. However, by sharing some of the regulatory pathways with AD, it has potential in treating the disease [75]. The final formulation had a size of approximately 100 nm with a dandelion morphology and no sign of aggregation. The mouse model was built by injecting Aβ, after which the mice were treated with 2 mg/kg of the formulation every other day for four weeks.

Zhang et al. [72] encapsulated cerium oxide nanocrystals into chemically functionalized NPs (1F12 antibody to specifically bind to all forms of Aβ_42_ and RVG29 to bind to receptors located on the surface of ECs and neurons for RMT) to scavenge ROS and prevent the formation of NFTs and Aβ plaques. The dual-functionalized loaded NP (RVG29- bMSNs@Ce 1F12) presented a size of ~110 nm, a ZP of 56.1 mV, and was well dispersed in water, indicating that it was stable. Despite the high positive value of ZP opposing risk to disrupt the BBB, no significant cytotoxicity was reported, either in vitro or in vivo. For brain targeting and Aβ plaque labeling, 10 mg/kg of Cy3-labeled RVG29- bMSNs@Ce-1F12 was administered in the mouse model, the same amount to evaluate Aβ clearance, but given weekly for 4 weeks.

Hou et al. [73] fabricated gold NPs decorated with a tripeptide antioxidant, glutathione, to protect tissues from ROS damage and enantioselectively prevent Aβ aggregation. In addition, this antioxidant allows BBB crossing by RMT. The vehicles were designed to possess diameters of 3.3 nm, which, despite being too low, and therefore risk being eliminated by the liver, succeed in their intent. For the in vivo studies, both L- and D-glutathione gold NPs were injected with a concentration of 25 mg/kg.

Hu et al. [74] encapsulated RES, a non-flavonoid found in red wine, almonds, and grapes, due to its potential therapeutic effects on AD. In vitro experiments confirmed its potential since it was able to reduce Aβ fibrils and even induce Aβ disaggregation, reshape toxic aggregates into non-toxic, promote autophagy and mitochondrial health by being a sirtuin 1 activator (reducing protein aggregation and oxidative damage), among other benefits [76,77]. Preclinical studies in AD model rats also demonstrated its anti-inflammatory and antioxidant effects and ability to decrease Aβ and plaque levels [77]. In addition, the NPs’ surface was decorated with lactoferrin to target lactoferrin receptors and ease brain internalization. The nanovehicles without RES presented a hydrodynamic diameter between 90 and 120 nm, a good dispersion, and a ZP of −44.0 ± 2.32 mV. The experiments in vivo were conducted by tail injection for 8 weeks.

### 4.2. Focused Ultrasound Combined with Nanoparticles

In contrast to the previously described strategy, which aims to enhance brain delivery by improving NP recognition, alternative strategies involve the transient and reversible opening of the BBB itself. Therefore, these approaches do not focus on altering NPs’ properties, but on temporarily modulating the structure and permeability of the BBB to facilitate NP penetration. Several techniques have been developed to achieve this, such as osmotic agents, lasers, and FUS-mediated BBB opening [78].

FUS is a safe, non-invasive, and reversible technique capable of temporarily disrupting the BBB. This method relies on low-frequency ultrasound waves, typically ranging from 2 to 18 MHz, which are delivered transcranially through sonication. FUS demonstrates considerable potential for enhancing drug delivery to the brain and treatment of neurological diseases [79].

However, effective BBB opening requires combining FUS with systemically administered MBs. MBs are a type of microsphere with diameters ranging from 0.5 to 10 μm that can be injected intravenously [79]. Therefore, FUS combined with MBs is a method that uses ultrasound waves directed at specific areas of the brain to cause oscillation of the MBs circulating in the blood, producing mechanical forces within blood vessels that temporarily and safely loosen the TJs of the BBB. Consequently, the BBB becomes more permeable, allowing therapeutic drugs to pass more easily from the bloodstream into the brain tissue [80,81].

Although no specific type of NPs has been reported to be most suitable for this technique, recent research has used PNPs, magnetic NPs, lipidic NPs, and inorganic NPs. Table 3 contains some examples currently being explored for drug delivery, tested in vitro and/or in vivo for the treatment of AD. The table highlights the diversity of nanocarrier matrices, the FUS conditions, the therapeutic agents being encapsulated, as well as administration routes, BBB opening strategy, test models used, and the main results obtained.

Nance et al. [82] produced PEG-coated PS or PLGA BPNs to penetrate the brain in regions where the BBB is disrupted by a combination of MRI-guided FUS and MBs. Initially, NPs were prepared with different commercially supplied sizes (40, 100 and 200 nm) for PEG and COOH coating, to determine the effect of particle size and surface chemistry on transport rates of modified particles in the mouse brain tissue. For the in vivo studies, a nano-injection device was set to inject $9.2\times 10^{-6}$ mL of particle solution at a rate of $23\times 10^{-6}$ mL/s in rat brain tissue slices.

Ogawa et al. [83] also used MB-assisted FUS BBB opening to investigate the intravenous delivery of encapsulated mRNA in lipid NPs (mRNA-LNPs) to the brain. The particles produced had a size of 93.1 ± 4.5 nm, a negative ZP, −3.61 ± 0.98 mV, and a PdI of 0.12 ± 0.08 with an encapsulation efficiency (EE) of mRNA of almost 100% (97.9 ± 0.50%). DdY mice were administered intravenously with 0.00125, 0.0025, or 0.005 mg (in terms of mRNA) mRNA-LNP and MBs (5 × 10^9^ particles/kg). Mice were then placed on the FUS apparatus.

Ohta et al. [84] investigated the effect of NP size on their delivery into the brain, assisted by FUS-induced BBB opening, using AuNPs of 3, 15, and 120 nm diameter. The synthesized AuNPs had average diameters of 3.7 ± 0.5, 14.4 ± 1.7, and 120 ± 7.2 nm, determined by TEM images and ZP of −28 to −38 mV. The particles were subsequently PEGylated, resulting in ZP values between –6 and –9 mV. In vivo studies were performed using a transcranial FUS exposure setup following the administration of MBs and the NPs (100 μL AuNPs (4 mg/mL)).

As investigated in the last example described, Liu et al. [85] also used MBs combined with FUS. In this case, Quercetin-modified sulfur NPs (Qc@SNPs) embedded into MBs (Qc@SNPs-MB) were synthesized. Qc has biological functions, such as anti-oxidation and anti-inflammation, and can also reduce neuronal apoptosis and Aβ content in the brain. In AD animal models, it can reduce neurotoxicity and increase cognitive levels. In this study, Qc modification was used to obtain stable sulfuric NPs. Qc@SNPs and SNPs particle sizes were 53 ± 15 nm and 45 ± 10 nm, respectively, and Qc@SNPs-MB had an average particle diameter of 2.1 ± 0.1 μm. It is also relevant to highlight that after the treatment with FUS, only substances with particle size below 100 nm were detected in Qc@SNPs-MB solution, which confirmed the destruction of the MB structure and release of Qc@SNPs from the shell. Regarding ZP, a value of −39.2 mV was obtained for regular MB, 44.3 mV after Qc@SNPs was embedded, and −38.4 mV after US treatment and washing. The ZP of the prepared NPs (SNPs and Qc@SNPs) also exceeded −30 mV, indicating that they were all stable. To assess the learning and cognitive abilities of C57BL/6 AD mice, the MWM test was performed after treatment. Mice were injected with Qc (5 mg/kg), SNPs MB (5 mg/kg), or Qc@SNPs-MB (NPs = 5 mg/kg), and for the last two cases, subjected to US exposure.

### 4.3. Stimuli-Responsive Nanoparticles

Another promising strategy to enhance drug delivery involves the use of stimulus-responsive NPs. These nanocarriers are designed to undergo controlled physicochemical transformations in response to specific internal or external stimuli. As a result, these systems may release drugs selectively, depending on specific biological signals in the body or environmental changes, therefore optimizing treatment efficacy and reducing systemic side effects. Internal stimuli include variations in pH, redox state, or increased levels of specific enzymes, while external triggers may involve temperature, magnetic fields, ultrasound, electric fields, or light [86]. Due to their ability to improve the targeting and controlled release of therapeutic agents, this type of system has gained a lot of interest over the years, making it attractive for targeted therapies in complex diseases, such as NDs. These diseases are often characterized by specific microenvironmental changes that provide a rationale for the use of stimuli-responsive nanocarriers [87]. Several types of stimuli-responsive nanocarriers have been explored for NDs, particularly AD.

The most developed intelligent delivery strategy is the pH-responsive systems. These systems are designed to consider the acidic features of pathological or intracellular environments (such as lysosomes and endosomes) to trigger carrier disassembly or swelling. Therefore, concentrating the treatment where it is needed, while protecting healthy regions from unwanted drug delivery. For their application, it is crucial to balance chemical stability with acid sensitivity. As such, these systems are designed to be stable in neutral pH (blood) but to break down or release the therapeutic agents in acidic pH. As a result, they will release their drug cargo only when they encounter these acidic environments. To achieve this, common strategies are employed, such as the addition of chemical bonds, hydrazone, Schiff base, or acid-labile ester bonds, that break apart in acidic conditions. Another strategy consists of the use of polymers like polyacrylic acid, which can change shape or structure in acidic conditions and adhere better to tissues (mucoadhesion), enhancing drug retention time at the target site and contributing to better penetration [87]. These systems have shown an increase in drug uptake and improvement in treatment effects in AD mouse models [88].

Additional responsive systems are the ROS-responsive NPs. In the brains of AD patients, oxidative stress plays a significant role, often leading to the excessive formation of free radicals, such as ROS. Three primary mechanisms contribute to this process: (i) macromolecule peroxidation, (ii) Aβ metal ion redox potential, and (iii) mitochondrial dysfunction [89]. Together, these processes lead to elevated ROS levels, which in turn cause lipid peroxidation, DNA damage, protein modifications, inflammatory cascades, and neuronal apoptosis, all of which accelerate disease progression. Since abnormally elevated ROS levels are a hallmark of AD pathology, ROS-responsive NPs have been developed, using sulfure, selenium, boronate, or thioether oxidation-sensitive linkers. Like pH-responsive NPs, these systems remain stable under physiological conditions but rapidly disassemble in high-ROS environments, allowing selective drug release at oxidative stress sites [87]. Future developments may focus on the combination of other stimuli and targeting ligands to improve the clinical application of these systems.

Another hallmark of AD pathology is the altered levels of specific enzymes in the brain, such as esterases, proteases, and phosphatases. For this reason, NPs can be designed specifically to be cleaved when in contact with these enzymes, destroying a bond that allows the release of the therapeutic agent. This strategy involves incorporating enzyme-cleavable peptides or chemical bonds into the nanocarriers, which are selectively recognized upon entering the lesion site and cleaved by target enzymes, allowing a precise drug release [87].

As previously mentioned, other types of stimuli-responsive nanocarriers are also being investigated. Regardless of the stimuli, these systems offer highly dynamic and adaptable vehicles for precise targeted drug delivery. They are engineered to exploit environmental triggers characteristic of NDs, such as pH, redox potential, enzymatic activity, and temperature, and therefore, provide localized and controlled drug release, with enhanced therapeutic effects and reduced systemic side effects. Each of the systems described presents several distinctive advantages. However, their triggering mechanisms present challenges due to their complexity. Dual- and multi-responsive nanocarriers represent a promising strategy to achieve higher targeting specificity. Nevertheless, the complexity of their design and the clinical translation remain obstacles to overcome [87].

Examples of stimuli-responsive NPs currently being explored for drug delivery for the treatment of AD are presented in Table 4.

Li et al. [90] maximized the full potential of the PBNPs. In addition to being ROS scavengers and inhibitors of Cu^2+^-induced Aβ monomers aggregation, they are suitable for photothermal therapy since they can convert the laser energy into thermal energy and enable localized thermal therapy, more specifically, by depolymerising Aβ fibrils. To tackle the reduced circulation time of these vehicles, they wrapped them with red blood cell membranes (RBCMs), which also have synergistic action since they have adsorption capacity for Cu^2+^. The PBNPs coated with the RBCMs had a size of 73.6 ± 2.5 nm, a ZP of −22.7 ± 1.3 mV, and a vesicle-like structure. For the cognitive assessment in the mice, the PBNPs/RBCMs were administered (0.2 mg/mL) every 3 days for 4 weeks and irradiated with NIR for 5 min, 24 h after injection. The same concentration of NPs labeled with a fluorescent dye was used for the in vivo distribution, and NIR was applied for 5 min at 0.5, 1, 2, 4, and 8 h.

Yang et al. [91] functionalized the mesoporous silica NPs (MSN) with AuNPs by linking with 4-carboxyphenylboronic acid. In the presence of high levels of H_2_O_2_, a typical environment that surrounds Aβ plaques, the arylboronic ester degrades. This process leads to the detachment of the AuNPs and the opening of the previously closed pores, allowing the loading molecule to diffuse through them. In this case, the encapsulated molecule is a metal chelator, clioquinol, that can inhibit metal-induced Aβ aggregation and block ROS formation. When released, the AuNPs can also contribute to inhibiting Aβ self-assembly. The clioquinol-loaded MSN-AuNPs (MSN-CQAuNPs) presented a size of 68.33 ± 2.78 nm, a ZP of +20.6 ± 0.45 mV with an elongated spherical morphology. For the in vivo experiments, the mice were administered with or without 5, 10, or 20 mg/kg of MSN-CQAuNPs every day until the 14th day, when they were sacrificed.

Yuan et al. [92] developed a dual-stimuli responsive nanoplatform by bonding NGF and RVG for neuroprotective and targeting effects, respectively. Under NIR radiation, ruthenium NPs can de-aggregate Aβ fibers, and in the presence of an environment characterized by high levels of ROS, the diselenide bond breaks, therefore resulting in the release of small ruthenium NPs and NGF. The nanocluster composed of ruthenium NPs dually functionalized presented an aggregated state with uniformly dispersed particles of ~80 nm that rapidly dispersed into singular particles in H_2_O_2_ presence. The mice were injected with the nanocluster at a concentration of 1 mg/kg.

Liu et al. [93] produced a ROS-responsive dendrimer with an Aβ peptide for brain internalization and p-Nrf2 due to its pivotal role in cellular redox homeostasis. The dendrimer with both modifications possessed a diameter of 21 nm and, most likely, a negative ZP. For the in vivo studies, 90 mice were equally divided into 5 groups and administered saline, p-Nrf2, PBP, APBP (Aβ peptide modified or unmodified nanoconjugates with p-Nrf2 linked—designated APBP or PBP, respectively) (normalized p-Nrf2 dosage at 5 mg/kg), respectively, once a week until the 37th week after birth. Afterwards, they were submitted to cognitive tests and progressively sacrificed for immunofluorescence staining and Western blot.

Lin et al. [94] focused on the crucial role of AChE, which catalyzes the hydrolysis of ACh, in the transmission of nerve impulses at the cholinergic synapses. For this, MSN functionalized with biocompatible SC_6_A and ACh were synthesized and loaded with tacrine (Tac). Tac is a small molecule used to inhibit the activity of AChE and results in improved cognition in clinical trials. The diameter obtained through DLS of the synthesized MSN-SC_6_A was 266 nm, indicating the possibility of MSN-SC_6_A being used in vivo. The efficacy of drug-loaded nanocarriers was investigated in C57BL6/J mice models divided into three groups: PBS, Tac, and MSN-Tac-SC_6_A. The mice were treated subcutaneously with Tac (2.5 mg/kg BW) or MSN-Tac-SC_6_A (the amount of Tac loaded in MSN-Tac-SC_6_A was 2.5 mg/kg BW). For the in vivo toxicity, the mice were treated subcutaneously with MSN-COOH (40 mg/kg BW) or MSN-SC_6_A (40 mg/kg BW).

Attia et al. [95] developed cationic nanoliposomes loaded with artesunate (ART-CLP). ART is an antimalarial drug with emerging neurotherapeutic potential that has shown promise in reducing amyloidosis in AD models. The plain CLPs produced had an average particle size of 83.20 ± 12.0 nm, a PdI of 0.08 ± 0.01, and a ZP of +20.15 ± 1.4. After encapsulation, the produced ART-CLPs showed an average particle size of 114.72 ± 13.1 nm, PdI of 0.1 ± 0.02, ZP of +16.65 ± 1.9 mV, and EE of 81.8 ± 7.8%, suggesting the ability of CLPs to encapsulate high concentrations of ART, which safeguards the improved therapeutic activity. TEM images displayed a uniform spherical morphology with minor aggregations of ART-CLP. For the in vivo studies performed, ART and ART-CLP were administered orally (40 mg/kg) for 60 days to adult male Swiss Albino mice. Following the injection via the intracerebroventricular route of streptozotocin (STZ) (3 mg/kg).

Geng et al. [96] designed conjugated polymer-based thermo-responsive micelles (CPMs) loaded with PF. In order to obtain the CPMs, two polymers were produced, PMO-b-PBM and POEG-b-PBM. PF was encapsulated in the micelles to generate ROS under white light irradiation. The average diameter of the CPMs produced was around 70 nm, and through a TEM image, CPMs displayed a spherical structure morphology. In this example, no in vivo studies were conducted.

Du et al. [97] designed UCNP conjugated with C_60_ and Aβ-target peptide KLVFF (UCNP@C_60_-pep) as a near-infrared-switchable nanoplatform. C_60_ presents a dual property: under UV/visible light, it can serve as a powerful ROS producer; however, in the dark, C_60_ acts as a ROS scavenger, due to its extremely delocalized π double bond system. TEM images showed the UCNPs had an average size of 30 nm. No additional information was provided regarding the physicochemical characteristics of the produced NPs, and no studies were performed in vivo.

### 4.4. Nanoparticles in Gene Therapy for AD Treatment

Gene therapy uses nucleic acids to treat genetic disorders [98]. However, nucleic acid agents in vivo are very susceptible to enzyme degradation in the bloodstream, present a low ability to cross the BBB, a potential to trigger immune responses and a low uptake efficiency. Nucleic acids hold the potential to inhibit target genes through complementary binding to target RNA (e.g., microRNAs (miRNAs)), enhance the expression of specific genes by inserting a functional gene copy to be expressed (e.g., mRNA) and even amplify, surpress, or correct gene expression, consequently resulting in gain or loss of function (e.g., clustered regularly interspaced short palindromic repeats (CRISPR)/Cas systems) [98,99]. Therefore, incorporating genetic material into NPs presents an appealing approach to overcome these challenges, since they can protect nucleic acid molecules as well as transport them to the desired locations [87,100].

In the context of AD, it was recently discovered that ApoE isoforms mediate several processes, such as cholesterol transport, neuronal signaling, Aβ clearance, and synaptic plasticity [98]. However, while ApoE2 is beneficial for preventing AD development, ApoE4 is a contributing factor to AD progression [101]. Consequently, correcting these dysfunctional ApoE isoforms using NPs with genetic material may represent a promising therapeutic strategy, particularly with the use of pDNA. Arora et al. [101] demonstrated efficient brain-targeted delivery of pApoE2 using GLUT-1-targeted, dual-functionalized liposomes, in in vitro (Transwell BBB model) and in vivo (C57BL/6 mice) studies. Results in vitro showed that dual-functionalized liposomes had 2 times greater transfection efficiency than plain and monofunctionalized liposomes. Following single-tail vein administration, the use of these liposomes to encapsulate the ApoE2/chitosan complex proved to significantly enhance the transport and transfection of the ApoE2 gene across the BBB in in vivo models, without any noticeable signs of toxicity. Transfection levels were evaluated using an ApoE ELISA kit. The results showed that dual-modified liposomes resulted in ∼2 times higher ApoE2 protein expression in the brain of mice compared to endogenous ApoE levels. Plain or monofunctionalized liposomes resulted in ∼1.25 times higher expression than the endogenous one.

Another marker in AD is the level of mRNA. On one hand, elevated levels of beta-site APP cleaving enzyme 1 (BACE1) mRNA can be detected in late stages of AD development, while reduced levels of BDNF mRNA can be reported in AD patients. Therefore, silencing BACE1 mRNA is a viable therapeutic approach, as well as enhancing BDNF mRNA expression [98]. In vitro and in vivo studies were performed to evaluate the efficacy of NPs systems. Lee et al. [102] developed liposomes encapsulating BACE1 siRNA, donepezil, or memantine, as well as their combination as a triple-drug therapy in treating AD. Results in vivo using female APP/PS1 homozygous transgenic mice showed induced short-term memory recovery, lowered levels of Aβ_40_ and Aβ_42_ peptides, reduction in BACE-1 mRNA, and inflammatory cytokine mRNA expression. Overall, the results highlighted the potential of this NP-based gene delivery system for AD therapy and its versatility for a range of CNS diseases. Li et al. [103] designed poly (β amino esters) (PBAE) NPs with BDNF mRNA encapsulated. Results demonstrated: (i) an increase in BDNF expression, confirmed by translation of the BDNF mRNA into protein in 293T cells and astrocytes and by functional release and neuronal activation in a transwell co-culture model; (ii) astrocyte-derived BDNF effectively protects neurons from Aβ-induced toxicity, restoring both cell viability and neuron proliferation in an in vitro transwell co-culture model; (iii) improved synaptic markers and behavioral outcomes in APP/PS1 double transgenic AD model mice.

Table 5 summarizes NP-based gene therapy approaches targeting specific genetic markers associated with AD.

Regardless of the genetic material encapsulated, different types of matrices have been used in this therapy. As previously mentioned, liposomes were used by Arora et al. [101] for the encapsulation of pApoE2; specifically, a liposomal matrix consisted of DOPE, DOTAP, cholesterol, and DSPE-PEG_2000_ lipids functionalized with cell-penetrating peptides and mannose ligands. Wang et al. [104] and Lee et al. [102] also use liposome matrices. On the other hand, Li et al. [103] and Zhou et al. [106] used PNPs, Park et al. [108] a peptide-based nanocomplex, and Lopez-Barbosa et al. [107], Wang et al. [109], and Carrillo-Jimenez et al. [110] used inorganic NPs.

### 4.5. Exosome-Mediated Drug Delivery

**Figure 4:**
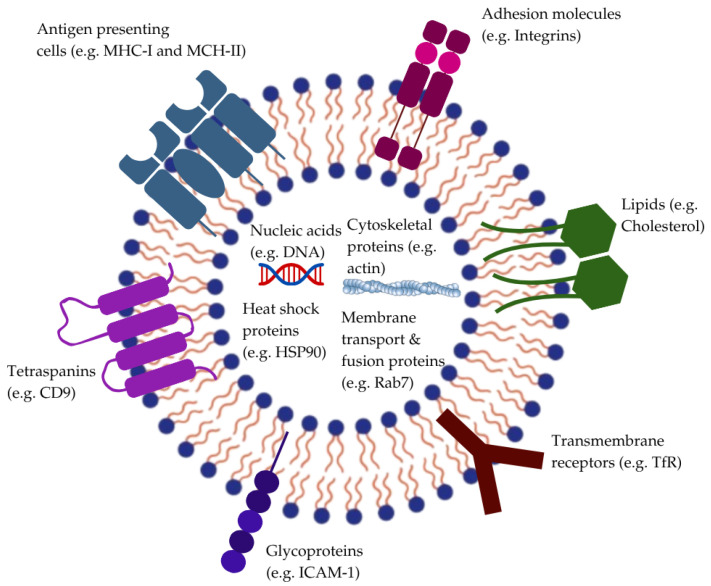
Exosome’s structure. Adapted from [112,113]. This image was created with the software Canvas (https://www.canva.com/).

Exosomes are extracellular vesicles with a diameter of around 30–150 nm, secreted by most cell types in the human body, and often found in physiological fluids such as blood, CSF, urine, and saliva [111,112]. Structurally, exosomes are composed of a lipid bilayer membrane responsible for protecting a diverse cargo of bioactive molecules like proteins, lipids, mRNA, and miRNA [112]. Additionally, they are enriched with specific proteins (e.g., tetraspanins, heat shock proteins) and their surface can present various receptors and adhesion molecules (Figure 4) [113].

Three main roles have been identified for exosomes: (i) to establish communication between cells and tissues by exchange of their intrinsic content; (ii) as diagnostic biomarkers; (iii) as DDS due to their properties (high stability, minimal immunogenicity, ease in crossing biological barriers, target specific cells or tissues, etc.) [111,112]. Their isolation for delivery purposes is a critical step, including methods such as sequential or density gradient ultracentrifugation, ultrafiltration, and size-exclusion chromatography, among others, each one with its respective advantages and limitations [113].

In the AD context, the role of exosomes is not fully clear. They seem to display a dual function in both expediting disease progression (e.g., triggering inflammation and oxidative stress) and slowing it down (Aβ and tau clearance) [111]. As DDS, several studies support their effectiveness in transporting therapeutic agents capable of reproducing beneficial outcomes. Huo et al. [114] produced silibinin-loaded macrophage-derived exosomes (Exo-Slb) by differential centrifugation combined with filtration and performed both in vitro and in vivo experiments. The exosomes’ size ranged from 30 to 120 nm. Exo-Slb injection resulted in higher accumulation of Slb in the hippocampus area of the brain, promoted its uptake in astrocytes, enhanced neuronal protection, increased the number of NeuN-positive cells, reversed Aβ-mediated damage, and exerted a better inhibitory effect on Aβ plaques. Cognitive assessment confirmed the shortest escape latency and alleviation of memory impairment in Exo-Slb-treated rats. Ultimately, both in vitro and in vivo tests showed a decrease in GFAP (glial fibrillary acidic protein) (thus maintaining astrocyte state in a resting position), a reduction in the inflammatory activity by decreasing the secretion of proinflammatory cytokines, downregulation of protein expression involved in NF-κB signaling, and the suppression of cell apoptosis.

Qi et al. [115] produced plasma-derived exosomes (by gradient ultracentrifugation) packed with Qc (Exo-Que) with an average of 150 nm and tested their performance in Okadaic acid (OA)-induced AD mice. Their administration was safe without systematic toxicity in vivo. Exo-Que significantly enhanced Qc’s pharmacokinetic profile, increased its accumulation in the brain, and improved the memory and spatial learning of SD rats. Moreover, it showed neuroprotective properties, suppressed the apoptosis of neurons in the hippocampus, and inhibited the formation of NFTs by limiting CDK5-mediated phosphorylation of tau protein, all these consequences derived from OA-injection. Ultimately, exosomes without Qc exerted a synergistic neuroprotection in rescuing memory deficits.

Wang et al. [116] isolated curcumin-loaded exosomes by ultracentrifugation with an average size of 117.4 nm and a ZP of −4.9 mV. Western plot analysis confirmed the inheritance of lymphocyte function-associated antigen 1 (LFA-1) from their parental cells, a protein that interacts with the endothelial intercellular adhesion molecule 1 (ICAM-1), used for BBB crossing. Enhanced brain penetration via RMT was also observed, with a 6.5 times higher brain concentration compared to free curcumin. Moreover, it enhanced curcumin solubility, stability, and bioavailability, and curcumin exhibited a slow and smooth release profile. No significant cytotoxicity was reported; in vivo, higher hippocampus accumulation was observed, and a reduction in OA-induced neuronal injury, cytotoxicity, and tau phosphorylation was confirmed. The MWM test revealed an amelioration of learning and memory abilities by presenting shorter escape latencies, spatially oriented swimming behavior, and more time in the targeted quadrant. Finally, na*ï*ve exosomes also reduced OA-induced consequences and inhibited microglia activation and neuronal cell apoptosis.

Table 6 provides key summarized aspects regarding the examples given above.

### 4.6. Magnetic Nanoparticles

Magnetic nanoparticles (MNPs) are a class of NPs that incorporate magnetic materials, such as iron, cobalt, and nickel, and exhibit magnetic behavior when exposed to magnetic fields. MNPs are composed of a magnetic core, most often magnetite, Fe_3_O_4_, or maghemite, α- or γ-Fe_2_O_3_, and a biocompatible surface [78]. Due to their unique superparamagnetic properties and diverse functionalities, such as magnetic guidance, heat generation under an alternating magnetic field, and image contrasts in magnetic-based medical imaging systems, these NPs are particularly interesting for CNS disease therapy [117]. The main applications of these NPs include magnetic hyperthermia, imaging contrast agents, theranostics, and magnetic drug targeting [118]. Therefore, studies have been conducted for the development of MNPs and their application in several neurological diseases, including AD.

The development of MNPs can follow different methods: chemical, based on chemical reactions (e.g., hydrothermal and solvothermal syntheses), physical, relying on the application of physical energy to break down larger solid material into nanosized particles (e.g., laser ablation and evaporation), and biological, resorting to the use of plant extracts and microorganisms. The choice of method relies on the specific biomedical application, since each technique enables the synthesis of MNPs with particular size, shape and physicochemical properties [119,120].

Studies using MNPs focus on three mechanisms: (i) modification with functional ligands, using peptides, antibodies and small molecules to enhance their transport across the BBB, as was previously described for other types of NPs; (ii) application of an external magnet field to guide MNPs as desired and disrupt temporarily the endothelial cell–cell junctions, facilitating their transport into the brain; and (iii) use of a low radiofrequency field which can also reversible and locally open the BBB [78]. Regardless of the mechanism, MNPs are a promising strategy for the treatment of diseases, as they can be controlled magnetically and target tissues affected by the disease. However, the success of the strategy also depends on the properties of the MNPs themselves, including size, magnetic properties, functionalization, and biocompatibility, as well as external factors [78].

MNPs have been extensively investigated in both in vitro and in vivo, to explore their ability to enhance brain targeting and drug delivery (Table 7). For instance, Ding et al. [121] produced multifunctional liposomal magnetic nanocarriers functionalized with Tf. Their performance was evaluated using a bi-compartmentalized transwell where primary human brain microvascular ECs and human astrocytes were grown to confluence on the upper chamber and underside of the lower chamber, respectively, with the presence and absence of an external magnetic force (EMF). The results showed a 50–100% increase in transmigration across the BBB when an EMF and Tf were used simultaneously, compared to their use in isolation, which indicated a positive synergetic effect. Nevertheless, their separate use still improved BBB delivery in comparison to traditional methods. Therefore, this study was able to produce MNPs with a significant potential in particle transmigration across the BBB and drug delivery into the brain. Gupta et al. [122] also used MNPs, intending to investigate a dual magnetic strategy to help the crossing of the BBB. Firstly, an EMF was used to enhance cellular association with the brain ECs, then an altering magnetic field (AMF) was applied to transiently loosen the TJs of the BBB. Results in vitro using transwell models developed by co-culturing murine brain ECs with astrocytes and in vivo, using C57BL/6 mice models, showed that a magnetic field allowed for the accumulation of NPs in the brain, caused a temporary opening of the BBB, and using both EMF and AMF led to more NPs reaching.

Although the existence of several studies highlighting the benefits of MNPs as effective vehicles to transport therapeutic agents through the BBB, their main application and contribution seem to mainly lie in their usage as imaging agents for the diagnosis of AD.

The content presented from Table 2, Table 3, Table 4, Table 5, Table 6 and Table 7, besides highlighting the NPs’ nature diversity, emphasizes the variety of loaded molecules, belonging to the most different classes, capable of reproducing therapeutic effects against AD.

Indeed, drug entrapment in NP-delivery systems allows their usage as therapeutic agents, contrary to their free form, in most cases, since their physicochemical properties preclude safe and effective travel across the body. Even the FDA and EMA-approved drugs to treat AD have been reported to manifest limitations (e.g., side effects, ineffective passage across the BBB, poor bioavailability, pharmacokinetics, and pharmacodynamics) that have been shown to be overcome by their employment in NPs [7]. Çinar et al. [126] compared the effect of donepezil-loaded PLGA-b-PEG NPs and free donepezil on adult Sprague-Dawley (SD) male rats previously injected with Aβ_25-35_ peptide. The results derived from cognitive and behavioral tests (MWM, novel object recognition (NOR), Elevated Plus Maze, and open field locomotor activity) showed that the rats treated with the drug-loaded nanosystem had the best improvement of short-term and spatial memory and hypo-anxiety-like behavior. After sacrifice, the AchE activity in the brain was determined, and the rats treated with the donepezil-NPs had the lowest percentage (excluding the naïve group), highlighting the effectiveness in returning the enzyme activity levels to normal.

In summary, all the advanced strategies presented either by engineering the design of the nanovehicles, encapsulating rational selected payload, or modulating the surrounding environment, intend to maximize the therapeutic response that reaches the brain. Each one of these is accompanied by its strengths and challenges. From the conducted research, the tailoring of specific ligands to the surface of nanoparticles seems to be the most studied strategy due to its simplicity and high targeting ability. Enhancing barrier permeability by FUS remains largely unexplored since it requires strict control of irradiation parameters and MB concentration. Therefore, inappropriate conditions could result in transient inflammatory responses, flash edema, and even intracerebral hemorrhage [78,80]. Innovative approaches include loading nucleic acids to correct dysregulations at the genomic level, although complex and potentially immunogenic. Repurposing exosomes for delivery is a compelling method (attributed to the properties entailed to them), but it is technically elaborate because of their isolation procedure. In addition, it requires treatment with the molecules aimed to be entrapped. Lastly, MNPs, despite being mostly employed as imaging agents, are very promising as they can be magnetically guided to the affected tissues. Their potential is confirmed by the fact that they are the only ones that received approval for human use [127].

## 5. Barriers to Clinical Translation of Nanoparticle-Based Therapies for Alzheimer’s Disease

Although the approval of over 50 NP-based therapies has occurred, only one is specifically brain-targeted (NanoTherm^®^, a magnetic-nanoparticle-based therapy intended for use in brain cancer), underscoring the struggle for effective clinical translation within this domain. The majority of clinical trials, in addition to being in early phases, are primarily focused on brain tumors, since NDs like AD express multiple and often interrelated pathogenic pathways hindering the discovery of universal therapeutic targets [127]. Several barriers explain the unsuccessful clinical translation of brain-targeted nanotherapeutics.

One concern, regarding the long-term safety of the therapy, is the chronic toxicity and immunogenicity of the nanoformulation. Their toxicity is directly modulated by their physicochemical properties (e.g., size, shape, surface area and charge) that must be properly addressed and determined. This toxicity can be manifested by the generation of ROS, disruption of cell membranes, DNA lesions, etc. [128]. Furthermore, it must be ensured that their approval is obtained in rigorous tests to confirm the safety of the NPs, such as long-term toxicological studies in animal models, monitoring of key biomarkers of neuroinflammation (e.g., TNF-α, IL-6, and other cytokines), immunotoxicity analyses, and cytotoxicity, among others, perceived as necessary. Altogether, they must guarantee that the repeated and long-term administration of the medicine poses no risk of neural damage, since even the slightest perturbation might produce irreversible damage [129].

Another major challenge is maintaining rigorous quality control in large-scale production, as it is an essential requirement to ensure consistency, reproducibility, and safety of the product. NPs used as DDS must have specific physicochemical properties that can affect immunogenicity, biodistribution, cellular uptake, and biocompatibility. As a result, inconsistencies during the manufacturing process may lead to unpredictable immune responses or altered safety profiles, making it challenging to ensure that every batch meets predefined quality and efficacy criteria. Additionally, biological safety assessments conducted throughout the production process are often insufficient, further complicating regulatory approval and delaying clinical application. To address these challenges, stricter quality control systems and standardized production processes need to be implemented to help minimize batch-to-batch variability, guarantee product reliability, and ensure safe and efficient industrial production [130].

The disparities in brain anatomy and complexity, metabolic processes, and age-related changes between animal models and humans explain why, despite their positive results in preclinical studies, they often fail to have therapeutic relevance in human trials. While essential to observe their effects in a living organism and study its pharmacokinetics, in vivo studies are robust and must be consistent and independent from interspecies variability [127]. From the literature found, mice and rats dominate as the most preferred animal models to study AD. However, they neither naturally nor experimentally induced develop AD. They simply replicate pathological features like Aβ accumulation, which often triggers other AD-typical symptoms like cognitive and memory impairments due to their interconnection. This limits the actual and real translation to humans; thus, developing realistic models featuring humanized characteristics represents a compelling strategy [131]. Recently, organ-on-a-chip platforms have gathered increased attention due to their capacity to emulate human physiology and homeostasis with enhanced reproducibility while reducing animal use in accordance with the 3Rs principle. More specifically, brain-on-a-chip technology, unlike conventional static in vitro models, incorporates endothelial barrier formation, fluid flow, and cell–cell interactions, enabling a more accurate assessment of NPs transport mechanisms and their effects in a realistic model that mimics key features of AD physiology [132].

Other limitations include off-target effects because of drug interaction with unwanted cells, disease heterogeneity demanding biomarker-driven patient classification for a personalized therapy, regulations, pricing, social considerations, and so on [127,129]. Ultimately, these limitations are often interligated (model heterogeneity impairs safety evaluation and increases off-target risks, scaled-up nanoformulations could have different physicochemical properties demanding new assessment of their safety and efficacy), adding to the complexity of this translation. Therefore, despite the efforts in developing strategies to enhance NP penetration and their ground-breaking results in preclinical studies, the gap to clinical translation onto human patients remains substantial. Moving forward, to expedite this translation, one can investigate theragnostic nanocarriers for dual action (therapeutic and diagnostic), standardize clinical studies, resort to artificial intelligence, co-deliver multiple therapeutic agents, improve nanoformulations, optimize drug-targeting, etc. [127,129].

## 6. Conclusions and Future Perspectives

The sustained increase in the AD prevalence, coupled with the absence of a cure, raises concern and highlights the need for the development of technologies capable of addressing this problem. In this paper, insights regarding the potential of nanotechnology-based DDS were explored, and, more importantly, how they emerge as a key to unlock effective and safe passage of therapeutic agents across the BBB, the most challenging obstacle in treating NDs.

Several types of NPs have been developed and tested as DDS. Their ability to navigate throughout the bloodstream and cross the BBB by themselves is limited; however, several strategies have been employed to enhance their circulation time, penetration, and performance. Surface modification, FUS-mediated NP delivery, and stimuli-responsive NPs are some of the strategies thoroughly explained in this review.

Despite the remarkable outcomes of these vehicles in preclinical settings, employing the most varied therapeutic molecules with multiple strategies, their clinical application in trials is nonexistent, which validates the need for increased effort in this area.

In 2021, after 19 years without any progression, the first immunotherapy-based (Aducanumab) was approved. Although later disapproved, it marked the first step in altering the course of the disease instead of only addressing its symptoms, opening a door of possibilities for new disease-modifying treatments. Other approaches being explored include the encapsulation of genetic material, NP-based combination therapy, and advanced types of NPs, such as MNPs and exosomes.

Looking forward, one can expect the continuous development of highly engineered NPs specially designed to safely and effectively target and deliver therapeutic molecules to the brain. For this purpose, a range of advanced strategies can be leveraged. The critical challenge lies in the clinical translation, where the most progress must be accomplished. Crucial to its advance is the standardization of the comprehensive NP characterization to ensure safety profiles and of the production for scalable synthesis, and the employment of these systems in novel models that better emulate AD’s physiology in humans (e.g., brain-on-a-chip platforms).

In conclusion, this paper underscores the importance of NP-based therapies for AD and the progress achieved in recent years. Nevertheless, continued research is still required to further optimize these systems and enable them to reach their full potential in revolutionizing the treatment landscape for AD.

## Figures and Tables

**Table 1:** Examples of ligands and stabilizers for NP functionalization.

**Pre-Transcytosis**				
**Ligand/Stabilizer**	**Category**	**Purpose**		**Ref.**
PEG	Polymer	“Stealth” molecule; prevents opsonization, phagocytosis, and capture by the reticuloendothelial system		[44]
PVA	Polymer	Non-ionic stabilizer that contributes to steric forces		[45]
P80/Tween 80	Polymer	Hydrophilic coating; protection against opsonization and reticuloendothelial system elimination		[46]
Pluronic F68	Polymer			[47]
Chitosan	Polysaccharide	Steric stabilization		[48]
**Transcytosis**				
**Ligand**	**Category**	**Uptake pathway**	**Target site**	**Ref.**
Tf	Glycoprotein	RMT	TfR	[49]
OX26	mAb	RMT	TfR	[50]
B6 peptide	Peptide	RMT	TfR	[51]
Insulin	Peptide hormone	RMT	IR	[52]
P80	Polymer	RMT	LDL receptor	[46]
Angiopep-2	CPP	RMT	LRP1	[53]
Folic acid	Synthetic form of the B9 vitamin	RMT	FR	[54]
GSH	Tripeptide	CMT	GSH transporter	[55]
Dipeptide phenylalanine	Dipeptide	CMT	LAT-1	[55]
Chitosan	Polysaccharide	AMT	- (no target site)	[48]
DMAB	Quaternary ammonium compound	AMT	-	[56]
**Post-transcytosis**				
**Ligand**	**Category**	**Target site**		**Ref.**
KLVFF	Peptide	Aβ_1–42_		[57]
DE2B4	mAb	Aβ plaque deposits		[50]
TPP	Lipophilic cation	Neuronal mitochondria		[58]
Mannose	Carbohydrate	Microglia		[59]
DMK6240	Aromatic heterocyclic amine	Hyperphosphorylated tau		[60]

CPP—cell-penetrating peptide; DMAB—didodecyldimethylammonium bromide; FR—folate receptor; GSH—glutathione; LAT-1—L-type amino acid transporter 1; LDL—low-density lipoprotein; LRP1—low-density lipoprotein receptor-related protein 1; mAb—monoclonal antibody; P80—Polysorbate 80; PEG—poly(ethylene glycol); TPP—triphenylphosphine cation.

**Table 2:** Examples of surface-modified nanocarrier formulations for AD.

Nanocarrier Matrix	Surface Functionalization	Loaded Molecule	Admin. Route	Test Model	Main Results	Ref.
Liposome (EPC, cholesterol, DSPE-PEG_2000_-MAL)	Tf	Osthole	ip. (10 mg/kg BW)	SH-SY5Y, HEK293T, hCMEC/D3 cells, APP/PS1 mice	Superior brain-targeting efficiency (Tf modification), existence of a sustained release profile, and higher circulation time (PEG decoration) Reduced risk of neuronal apoptosis, oxidative stress, and neuroinflammation Diminished Aβ deposits, downregulation of Aβ_1-42_ (most prone to form aggregates), and normal neuronal and mitochondrial morphology Improved cognitive function	[49]
Liposome (DSPE-PEG-MAN (4%), DSPE-PEG-CPP (4%), DOPE (45%), DOTAP (45%), and cholesterol (2%))	MAN and CPPs	pBDNF	iv. (0.4 mg/kg BW)	Transgenic APP/PS1 mice	Liposomal formulations biocompatible both in vitro and in vivo Enhanced BDNF expression by ~2 times, resulting in a reduction in toxic Aβ peptides Plaque load was reduced, whereas synaptic proteins (synaptophysin and PSD-95) increased No adverse effects were observed, indicating a safe and effective strategy to rescue AD pathology	[65]
TR@CPL (soybean phospholipids, cholesterol, DSPE-PEG)	CCR2-overexpressing and platelet membranes	Rapamycin and TPPU	iv.	HEK293T, Neuro2a, HT22, SH-SY5Y and BV2 cell lines, 5xFAD mice	Rescue cell death and the ability to target inflammatory lesions Low biological toxicity Ameliorated cell death caused by excessive Aβ_42_ Improved cognition Promoted autophagy (Aβ and tau clearance) and reduced APP expression Reduce the number of glial cell aggregates (alleviate neuroinflammation) and amyloid plaque burden	[66]
Hydroxyl dendrimer	D-45113	- (no loaded molecule)	ip. (200 mg/kg BW)	5xFAD mouse model	Ability to cross the BBB Significant reduction in Aβ Stark reduction in the number of microglia and microglia-plaque association in the subiculum and somatosensory cortex Downregulation in microglial, inflammatory, and synaptic gene expression Therapeutic potential to cross a slightly impaired BBB and specifically target and modulate plaque-associated microglia in AD while leaving other cell types unaffected	[67]
Prussian blue/Gd-PAMAM	Angiopep-2	-	ip. (0.0004 mg)	BV-2 microglia cell line APP/PS1 transgenic mice (C57BL/6)	Increased penetration across the BBB when Angiopep-2 is conjugated to the NPs Reduced oxidation products, inflammatory cytokines, and consequently, neurotoxic Aβ aggregation Improved spatial learning, memory, and brain function	[53]
S@A-NPs (PLGA-PLL sphere)	Angiopep-2	Simvastatin	iv. (100 mg/kg BW)	bEND.3 mouse cell line of BMECs APP/PS1 transgenic mice	Upregulation of endothelial LRP1 expression Alleviated BBB damage, reduced Aβ burden, and attenuated neuroinflammation, neurodegeneration, and cognitive deficits Suppression of amyloidogenic APP cleavage Enhanced Aβ transport across the BBB from brain to blood	[68]
Cationic-NLCs (stearylamine as the solid lipid and capryol PMGC as the liquid)	Tf	Rapamycin	ih. (3.5 mg/kg BW)	Adult male Wistar albino rats	High entrapment efficiency and low cellular toxicity Capacity to penetrate the BBB in a TfR-dependent manner Eliminate the immunosuppressive effect of rapamycin Improved cognitive function by abolishment of Aβ-induced memory impairment and the ability to reverse those effects Attenuated Aβ-induced neuronal damage (fewer necrotic neuronal cells) Decrease in Aβ and Aβ-induced oxidative stress Modulation of mTOR activity and autophagy level Protective effect against apoptotic cell death	[69]
P[(OEG)_10_MA]_20_-PDPA_120_ polymersome	Angiopep-2	-	iv.	APP/PS1 mice	41% Aβ clearance after a few hours by reactivating the PACSIN2 pathway (instead of the Rab5 that accelerates amyloidogenic aggregation) 78% recovery of LRP1-CD31 colonization, suggesting BBB restoration Improved performance in cognitive tests	[70]
Mesoporous nano-selenium	Tf	Metformin	iv. (2 mg/kg BW)	SH-SY5Y, BV-2, bEnd.3 and PC12 cells C57BL/6 mice	Inhibit Aβ aggregation and degradation of Aβ fibrils, reduced Aβ-induced neurotoxicity and oxidative stress (by scavenging abnormal ROS and reactive nitrogen species), reverse the proinflammatory activation of microglia and astrocytes (by shifting the phenotype from M1 to M2) Efficient passage across the BBB, low cytotoxicity, and high biocompatibility Effective in rescuing the cognitive and memory abilities (improvement of memory and object recognition)	[71]
Mesoporous silica NPs	1F12 antibody RVG29	Ultra-small cerium oxide nanocrystals	iv. (10 mg/kg BW)	SH-SY5Y and BV2 cell lines APP/PS1 transgenic mice	Inhibition of Aβ_42_ aggregation and promotion of insoluble fibril depolymerization Peripheral capture and clearance of Aβ_42_ via intestinal metabolism and blood circulation ROS scavenging and reduced oxidative stress Attenuation of microgliosis in vivo Improvement of olfactory and cognitive impairments	[72]
AuNPs	Chiral L- and D-glutathione	-	iv. (25 mg/kg BW)	Kun Ming mice	Prevent Aβ aggregation by adsorbing peptide monomers on their curved surfaces Ability to cross the BBB through simple intravenous injection Decreased Aβ plaque deposition in the brain and reduced memory deficits Exhibit good biosafety and a rather rapid clearance from the body	[73]
Hollow-structured manganese-doped cerium dioxide NPs	Lf	RES	iv.	SH-SY5Y and bEnd.3 cells 5×FAD mice	Slow-release rate, improved RES bioavailability, and no significant toxic side effects Ability to penetrate the BBB and aggregate at neurons and microglia Antioxidant capacity in alleviating oxidative stress generated by Aβ induction through the Nrf2-HO-1 pathway Ability to scavenge ROS, inhibit Aβ aggregation, and facilitate neuronal recovery Improved performance and nesting abilities	[74]

APP—amyloid precursor protein; BDNF—brain-derived neurotrophic factor; CCR2—chemokine (C–C motif) receptor 2; CD31—platelet endothelial cell adhesion molecule-1; CPP- cell penetrationg peptide; D-45113—colony stimulating factor-1 receptor (CSF1R) inhibitor; DOPE—dioleoylphosphatidylethanolamine; DSPE-PEG_2000_-MAL—1.2-distearoyl-sn-glycero-3-phosphoethanolamine-N [carboxy (polyethylene glycol)-2000]; EPC—egg yolk phosphatidylcholine; iv.—intravenous, ip.—intraperitoneal; ih.—intrahippocampal; MA—methacrylate; MAN—mannose; DOTAP—dioleoyltrimethylammonium propane; mTOR—mammalian target of rapamycin; Nrf2-HO-1—nuclear factor erythroid 2–related factor 2—heme oxygenase-1; OEG—oligo(ethylene glycol); PACSIN2—protein kinase C and casein kinase substrate in neurons 2; PAMAM—polyamidoamine; PDPA—poly(2-(diisopropylamino)ethyl methacrylate); PLL—poly (ε-carbobenzoxyl-L-lysine); PSD-95—postsynaptic density protein 95; RES—resveratrol; TPPU—1-trifluoromethoxyphenyl-3-(1-14 propionylpiperidin-4-yl)urea; ROS—reactive oxygen species; RVG29—rabies virus glycoprotein 29; TR@CPL—platelet membrane hybrid double drug-loaded liposomes; S@A-NPs—simvastatin-loaded Angiopep-2-anchored nanoparticles.

**Table 3:** Examples of FUS-combined NPs for AD.

**Nanocarrier Matrix**	FUS Conditions	Loaded Molecule	Admin. Route	BBB Opening Strategy	Test Model	Results	Ref.
BPNs (PEG-PLGA and PS-PEG NPs)	Two separate sonications were performed, one per hemisphere, using the interleaved sonication mode. Pressures ranged from 2.0 to 0.4 MPa	- (no loaded molecule)	iv. $(9.2\times 10^{-6}$ mL)	MRgFUS with intravascular MBs	Female CF-1 mice (Harlan)	Safe, pressure-dependent delivery of BPNs to the brain parenchyma, where BBB disruption was implemented 10-fold slower diffuse rate of PEGylated BPNs into the brain parenchyma, allowing for a sustained drug delivery profile	[82]
Lipid NPs (COATSOME SS-OP, DOPC, cholesterol and DMG-PEG_2000_)	FUS was irradiated to the right striatum: frequency, 3 MHz; intensity, 0.5–1500 W/cm^2^; duration, 60 s; duty cycle, 10%	mRNA	iv. (0.00125, 0.0025, or 0.005 mg)	MBs FUS-induced	Male ddY mice	Enhanced exogenous protein expression in the regions where FUS irradiation and microbubbles were both administered (increase to 7.1 pg/mg) No hemorrhage or edema was found in mice Lower mRNA-LNP dosage is sufficient (no increase in protein expression with higher dosages)	[83]
PEG-AuNPs	FUS exposure through a water bath for 40 s, with a burst length of 0.01 s, a repetition frequency of $1\times 10^{-6}$ MHz and a resonant frequency and peak-positive pressure of 1 MHz and 0.088 MPa, respectively Transcranial FUS exposure	-	iv. (0.4 mg)	FUS combined with sozanoid MBs	Human umbilical vein cell bEND.3 cells ICR mice	FUS exposure resulted in small gaps (less than the sub-micron scale), and afterwards BBB returned to its original state Good biocompatibility of the NPs Existence of an optimum size of NPs because of the competition between permeation through the gaps and excretion by the system (3, 15, and 120 nm AuNPs showed increased brain accumulation of 3.1, 18.2, and 5.4 times)	[84]
Sulfur NPs	US exposure for 600 s after injection (sound pressure about 1 MPa)	-	iv. (5 mg/kg BW)	FUS combined with MBs	SH-SY5Y cells APP/PS1 mice	Inducement of BBB opening and entry of Qc@SNPs into the brain Reduction in neuronal apoptosis, inflammatory response, Aβ content, calcium homeostasis imbalance, and oxidative stress Protection of nerve cells Improvement in the learning and memory abilities (MWM test) without obvious side effects	[85]

BPNs—brain-penetrating NPs; DMG-PEG_2000—_1,2-dimyristoyl-sn-glycerol-(methoxy(polyethylene glycol)-2000; DOPC—dioleoylphosphatidylcholine; FUS-Focused Ultrasound; LNP—lipid NP; MWM—Morris water maze; MRgFUS—transcranial magnetic resonance-guided FUS; PS—polystyrene; mRNA—messenger RNA; Qc—quercitin.

**Table 4:** Examples of stimuli-responsive NPs for AD.

Nanocarrier Matrix	Surface Functionalization /Stimuli-Responsive Material	Loaded Molecule	Admin. Route	Stimuli	Test Model	Results	Ref.
PBNPs	RBC/PBNPs	- (no loaded molecule)	iv. (0.2 mg/mL)	NIR (808 nm, 1 W/cm^2^)	bEnd.3 brain endothelial cells SH-SY5Y neuronal cells BV2 cells APP/PS1 mice	Excellent photothermal ability that can temporarily open the BBB, enhance the transmission efficiency of PB/RBC across the BBB, and depolymerise the formed Aβ deposits Successful inhibition of Cu^2+^-induced Aβ monomer aggregation, elimination of the deposition of Aβ plaques and reduction in the ROS levels Improve the quality of mitochondria, restore the phagocytic function of microglia and alleviate neuroinflammation Significant amelioration of cognitive and learning deficits	[90]
Mesoporous silica NPs	AuNPs/arylboronic ester	Clioquinol	iv. (5, 10, or 20 mg/kg BW)	H_2_O_2_	PC12 and bEnd.3 cells Female ICR mice	Enhanced inhibition of Cu^2+^- induced Aβ_40_ aggregation Neuroprotection of PC12 cells Additional suppression of Aβ self-assembly mediated by AuNPs Reduced membrane damage, microtubule disruption, and ROS-mediated apoptosis Controlled and stimuli-responsive release of clioquinol High BBB permeability, effective cellular uptake, selective intracellular release of metal chelators, and good biocompatibility	[91]
Ruthenium NPs	NGF and RV/GRuthenium NPs (NIR) and –Se–Se-bond (ROS)	-	iv. (1 mg/kg BW)	ROS and NIR (750 nm, 1 W/cm^2^)	SH-SY5Y cells bEnd3 cells APP/PS1 Tg mice	Stable nanosystem in a physiological environment and good biocompatibility Quick response to ROS Energy and time-dependent absorption into cells and ability to be degraded by ROS Under NIR irradiation, inhibition of Aβ accumulation and disaggregation of preformed Aβ fibrils Good neurocyte protection (inhibits Aβ-induced cytotoxicity) RVG-modification allowed for active brain targeting and higher brain accumulation Enhanced nesting ability of mice and inhibition of Aβ plaque formation	[92]
Dendrimer (MeO-PEG-NH_2_, alkynyl phenylboronic ester)	KLVFFAED (designated Aβ peptide) and p-Nrf2/PEG-based phenylboronic dendrimer	-	iv. (5 mg/kg BW)	ROS	SH-SY5Y cells, BV2 cells, and brain capillary endothelial cells APP/PS1 Tg mice	Aβ peptide modification enhanced cellular uptake and allowed for passage across the BBB Activation of glial cells—polarize M1-like microglia to an M2-like phenotype Antioxidant and anti-apoptotic capacity ROS scavenger, restores the antioxidant capacity by activating the Nrf2-dependent antioxidant pathway Facilitated Aβ clearance and inhibited its aggregation Attenuated cognitive and memory impairments Biocompatible	[93]
Mesoporous silica NPs	ACh and SC_6_A/complex ACh and SC_6_A	Tacrine	s.c. (2.5 or 40 mg/kg BW)	Enzyme (AChE)	Bel7402 cells C57BL6/J mice	Controlled release profile of the AChE, allowing for a sustained release Reduced hepatotoxicity due to a controlled-release system, and only happening in disease conditions Biocompatible	[94]
Cationic lipid NPs (DPPC, DOTAP, Cholesterol)	-/No information provided	Artesunate	Oral (40 mg/kg BW)	pH	Adult male Swiss Albino mice	Sensitive to acidic pH—selective diffusion of the loaded drug in Alzheimer’s tissues Inhibition of key inflammatory pathways (e.g., by curbing caspase 11 and GSDMD-N levels) Mitigation of AD markers (Aβ and tau levels) Enhanced memory efficiency	[95]
Polymer-based micelles (PMO-b-PBM and POEG-b-PBM)	-/PMO	PF	-	T	PC-12 cells	Inhibition of Aβ aggregation through capture of Aβ species, fibrillation process, and transformation from α-helical to β-sheet structure Ability to disaggregate the aggregates by ROS generation Biocompatible Enhanced cell viability by suppressing Aβ aggregation and associated cytotoxicity	[96]
NaGdF_4_:Yb/Er/Tm UCNPs	Fullerene (C_60_) and KLVFF (Aβ target peptide)	-	feeding	NIR, VIS/UV light	PC12-Aβ cells *C. elegans* CL2006	Under NIR, ROS production hinders Aβ aggregation and mitigates its cytotoxicity, whilst in the dark, it has ROS-quenching abilities contributing to an increase in the lifespan of the *C. elegans* worms	[97]

ACh—acetylcholine; AChE—acetylcholinesterase; *C. elegans*—*Caenorhabditis elegans* DPPC—1,2-dipalmitoyl-sn-glycero-3-phosphocholine; DOTAP—dioleoyltrimethylammonium propane; GSDMD-N—N-terminal fragment of gasdermin D; NGF—nerve growth factor; NIR—near-infrared; p-Nrf2—phosphorylated nuclear factor (erythroid-derived 2)-like 2; PBNPs—Prussian blue NPs; PB/RBC—peripheral blood/red blood cells; PBM—poly(butyl methacrylate); PF—polyfluorene conjugated polymers; PMO—poly-2-(2-methoxy ethoxy)-ethyl methacrylate-cooligo(ethylene glycol) methacrylate; POEG—polyoligo(ethylene glycol) methacrylates; ROS—reactive oxygen species; SC_6_A—sulfonatocalix[6]arene; s.c.—subcutaneous application; T—temperature; Tg—transgenic; UCNPs—upconversion NPs; VIS/UV—visible/ultraviolet.

**Table 5:** NPs-based gene therapy approaches targeting genetic markers associated with AD.

Genetic Marker	Biological Function in AD	Genetic Material Loaded in NPs	Gene Therapy Approach	Ref.
ApoE isoforms (ApoE4 and ApoE2)	Cholesterol transport, neuronal signaling, amyloid-β clearance and synaptic plasticity	pDNA encoding ApoE2 (pApoE2)	Gene expression to increase ApoE2 levels and counteract ApoE4-associated pathological effects	[101,104]
BACE1	Key enzyme involved in Aβ production	BACE-1 siRNA	Gene silencing to reduce BACE1 expression and Aβ production	[102,105,106,107]
		CRISPR-Cas9		[108]
BDNF	Neuronal survival, synaptic plasticity, and memory	BDNF mRNA	Gene expression to restore reduced BDNF levels observed in AD	[103]
		Plasmid encoding BDNF		[65]
APP	Precursor of Aβ peptides	CRISPR	Gene editing of the AD-related gene and reduction in the expression of APP	[109]
TREM2	Involved in regulating microglial inflammatory responses and associated with AD pathology and impairment	TREM2 siRNA	Gene silencing to reduce TREM2 levels	[110]

ApoEA—polipoprotein E; APP-Amyloid Precursor Protein; BACE— Beta-site APP-cleaving enzyme 1; BDNF-Brain-Derived Neurotrophic Factor; siRNA-Small interfiring RNA; NPs-nnoparticles; TREM2—triggering receptor expressed on myeloid cells 2.

**Table 6:** Examples of exosome-mediated drug delivery systems.

Exosome Nature	Loaded Molecule (Internalization)	Admin. Route	Test Model	Main Findings	Ref.
Macrophage-derived	Silibinin (treated with the macrophage cells)	iv.	SH-SY5Y neuroblastoma cells and astrocyte cell line HA-1800 SD rats	Reduction in Aβ_1-42_ deposition, astrocyte deactivation and alleviation of neuronal injury	[114]
Plasma-derived	Qc (by shaking and ultrasonic incubation with exosomes)	iv.	OA-induced SD mice	Safe, improved drug bioavailability and ameliorated cognitive dysfunction	[115]
Mouse macrophage cells-derived (RAW 264.7 cell line)	Curcumin (treated with the macrophage cells)	iv. ip.	hCMEC/D3 cells, SD rats and OA-induced C57BL/6 mice	Safe, efficient BBB penetration, amelioration of learning and memory impairments and neuroprotective effects	[116]

BBB-Blood-brain barrier; OA-osteoarthritis; Qc-quercetin; SD-Sprague Dawley.

**Table 7:** Examples of MNPs for AD.

Type of MNPs	Loaded Molecule	Results	Ref.
PEGylated MNPs	siRNA and co-immobilized with OmpA	Effective BACE1 silencing in HFF-1 cells, without any presence of cytotoxic effect Biocompatible Co-immobilization improved cellular internalization and endosomal escape	[107]
Iron Oxide NPs	MSC	Higher brain retention under magnetic guidance Enhanced therapeutic molecule expression of MSCs Significant reduction in Aβ plaque burden and improvement of cognitive function in vivo models	[123]
Iron Oxide NPs conjugated with D-TLKIVWC peptide (7-DP)	-	Conjugation of 7-DP to MNPs enables transport across the BBB Retention of the inhibitory and fibril-fragmenting properties of free 7-DP Reduced tau-induced cell toxicity in vitro Reverse neurological deficits in vivo	[124]
Dextran-coated Fe_3_O_4_ MNPs	Osmotin	Attenuation of Aβ_1–42_–induced synaptic deficits and memory impairment Reduction in Aβ accumulation and BACE-1 expression Inhibition of tau hyperphosphorylation	[125]
Iron oxide	siRNA (siTREM2 and siCD33)	Efficient knockdown of TREM2 and CD33 expression in vivo Low toxicity and non-priming effect on the inflammatory response	[110]

BACE—Beta-site APP-cleaving enzyme 1; HFF-human foreskin fibroblast cell line; MNPs-Magnetic nanoparticles; MSC—Mesenchymal stem cell; Nps-nanoparticles; OmpA-Outer Membrane Protein A.

## Data Availability

No new data were created or analyzed in this study. Data sharing is not applicable to this article.
